# Sex Differences in Presynaptic Density and Neurogenesis in Middle-Aged ApoE4 and ApoE Knockout Mice

**DOI:** 10.1155/2013/531326

**Published:** 2013-01-27

**Authors:** A. Rijpma, D. Jansen, I. A. C. Arnoldussen, X. T. Fang, M. Wiesmann, M. P. C. Mutsaers, P. J. Dederen, C. I. F. Janssen, A. J. Kiliaan

**Affiliations:** Department of Anatomy, Donders Centre for Neuroscience, Radboud University Nijmegen Medical Centre, Geert Grooteplein 21, 6525 EZ Nijmegen, The Netherlands

## Abstract

Atherosclerosis and apolipoprotein E *ε*4 (APOE4) genotype are risk factors for Alzheimer's disease (AD) and cardiovascular disease (CVD). Sex differences exist in prevalence and manifestation of both diseases. We investigated sex differences respective to aging, focusing on cognitive parameters in apoE4 and apoE knockout (ko) mouse models of AD and CVD. Presynaptic density and neurogenesis were investigated immunohistochemically in male and female apoE4, apoE ko, and wild-type mice. Middle-aged female apoE4 mice showed decreased presynaptic density in the inner molecular layer of the dentate gyrus of the hippocampus. Middle-aged female apoE ko mice showed a trend towards increased neurogenesis in the hippocampus compared with wild-type mice. No differences in these parameters could be observed in middle-aged male mice. Specific harmful interactions between apoE4 and estrogen could be responsible for decreased presynaptic density in female apoE4 mice. The trend of increased neurogenesis found in female apoE ko mice supports previous studies suggesting that temporarily increased amount of synaptic contacts and/or neurogenesis is a compensatory mechanism for synaptic failure. To our knowledge, no other studies investigating presynaptic density in aging female apoE4 or apoE ko mice are available. Sex-specific differences between APOE genotypes could account for some sex differences in AD and CVD.

## 1. Introduction

Western society is currently faced with an increasing incidence of vascular diseases such as hypercholesterolemia and atherosclerosis, mainly as a consequence of unhealthy lifestyle habits, an increase in obesity, and an aging population. Vascular diseases and obesity are risk factors for disorders that affect cognitive function such as diabetes mellitus, stroke, vascular dementia, and Alzheimer's disease (AD) [1, 2]. Sex differences exist in both AD and cardiovascular disease (CVD). While women have a higher risk for AD, men are generally more affected by CVD [3]. For example, men are more prone to develop high serum cholesterol levels at a younger age than (premenopausal) women [4, 5]. Most of these sex differences disappear, however, when women reach menopause, when they equal and even surpass men in the prevalence of CVD [3, 6]. However, differences in the manifestation of CVD remain. For example, while men have thicker atherosclerotic plaques in the large coronary arteries, women tend to have more diffuse plaques that also impair the smaller microvasculature [4, 5, 7]. 

 One factor influencing the prevalence of both vascular and Alzheimer's disease is the apolipoprotein E (APOE) genotype. The APOE genotype is a genetic risk factor for sporadic or late-onset AD. The risk for AD is increased in carriers of the *ε*4 allele [8, 9], and the age of onset is decreased [8, 10] compared with *ε*2 and *ε*3 allele carriers. Furthermore, in the nondemented elderly population and in healthy middle-aged people, the *ε*4 allele is also associated with memory decline [11–14]. The APOE gene encodes for the apolipoprotein E protein (apoE), which has several roles in the body and in the brain. These include anti-inflammatory and antioxidant effects [15–17] and cholesterol transport [18]. The apoE4 isoform is a poorer functioning cholesterol transporter, which contributes to hypercholesterolemia [19, 20], which in turn causes atherosclerosis. In addition, it has been shown in the brain that less apoE3 than apoE4 is needed for similar sized lipid particles, suggesting an impaired or less effective delivery of cholesterol to neurons [21]. Furthermore, apoE is involved in the clearance of amyloid-beta (A*β*) from the brain across the blood brain barrier [22]. Increased levels of A*β* in the brain lead to an increased risk of AD, and A*β* accumulation in blood vessel walls may lead to cerebral amyloid angiopathy. Obstruction of blood vessels by atherosclerosis or cerebral amyloid angiopathy can affect cognition by causing cerebral hypoperfusion. This reduces neuronal protein synthesis, which is important for synaptic plasticity [23]. Furthermore, it has been shown that cerebral blood flow (CBF) is reduced in AD patients [24], and CBF reductions are predictive of conversion to AD in patients with mild cognitive impairment [25]. 

As with CVD and AD, the APOE genotype has differential effects between the sexes. The increased risk for AD and cognitive deficits in the nondemented population in *ε*4 carriers is higher in women [26–28]. Furthermore, female apoE4 mice show spatial memory deficits [29, 30]. Estrogen is one factor that interacts with apoE, and as such could explain these effects. For example, hormone replacement therapy in menopausal women may not be beneficial for cognition in carriers of an *ε*4 allele [31, 32]. Additionally, whereas estradiol facilitates neurite outgrowth in the presence of apoE2 and apoE3, it does not do so in the presence of apoE4 [33]. In addition, brain apoE levels can be affected by pharmacological estrogen treatment, and this effect is brain region-specific, most likely due to the differential distribution of estrogen receptor subtypes [34]. 

The aim of the current study was to investigate sex differences in apoE, focusing on parameters that are important for cognitive functioning. Therefore, we studied presynaptic density and neurogenesis in male and female apoE4 and apoE knockout mice, which are models of AD and vascular disease. 

## 2. Experimental Procedures

### 2.1. Animals

Homozygous apoE-deficient (B6.129P2-Apoe^tm1Unc^/J) mice were originally obtained from Jackson Laboratories (Bar Harbor, ME, USA) and subsequently bred in the Central Animal Laboratory (CDL; Radboud University Nijmegen Medical Centre, RUNMC). The background strain for these apoE knockout (apoE ko) mice is C57BL6/J. Homozygous human APOE4 knockin mice were originally obtained from Taconic Transgenic Models (Hudson, NY, USA) and subsequently bred in the Central Animal Laboratory (RUNMC). In this strain, the murine ApoE gene is replaced with the human APOE4 alleles (4/4), after which the strain is backcrossed to C57BL6/J mice. C57BL6/J wild-type (WT) mice were originally obtained from Harlan Laboratories, Inc. (Horst, The Netherlands) and subsequently bred in the CDL (RUNMC). 

In total, 22 male mice and 21 female mice of the 3 different genotypes (WT, apoE4, and apoE ko) aged 9–15 months (males: 9–12 months, M: 10.7; females: 12–15 months, M: 13.2) were used in this study. The mice were housed in standard cages (Makrolon type 3, 42.5 × 26.5 × 15.5 cm, with maximum 11 animals per cage) at 21°C on a 12 h light/dark cycle (lights on at 7 a.m.) and were fed rodent lab chow. Water and food were provided ad libitum. There were no differences in brain or body weight between the genotypes in males or females.

The experiments were performed according to Dutch federal regulations for animal protection and were approved by the Veterinary Authority of the Radboud University Nijmegen Medical Centre (permit numbers 11-090 and 2008-172).

### 2.2. Tissue Preparation

Mice were anaesthetised with isoflurane (3-3.5% in a mixture of oxygen and N_2_O (2 : 1)) and transcardially perfused with phosphate buffered saline (0.1 M PBS) and with 4% paraformaldehyde. The brains were removed immediately after perfusion fixation, postfixed overnight in 4% paraformaldehyde at 4°C, and transferred the next day to 0.1 M PBS containing 1% sodium azide. The brains were placed in 30% sucrose in 0.1 M phosphate buffer for 24 hours before cutting 40 *μ*m coronal sections on a sliding microtome (Microm HM 440, Walldorf, Germany) equipped with an object table for freeze-sectioning at −60°C. Sections were divided into 6 complete series (1 out of every 6 sections) while cutting. The sections were stored in 0.1 M PBS with 1% sodium azide at 4°C until they were used for immunohistochemistry.

### 2.3. Immunohistochemistry

All staining was carried out according to standard protocols, and all steps were performed at room temperature on a shaker table. The staining was performed in separate sessions; male apoE4, apoE ko, and WT mice were stained in one group, and female apoE4, apoE ko, and WT mice were stained in another group. Before each staining session, a test staining was performed to determine the optimal antibody concentration. Free-floating brain sections were first rinsed (rinsing of sections was always done with 0.1 M PBS) and endogenous peroxidase was blocked with 0.3% H_2_O_2_ in 0.1 M PBS. The sections were rinsed again and preincubated with 0.1 M PBS-BT (0.1 M PBS with 1% Bovine Serum Albumin and 0.3% Triton-X-100). The sections were then incubated overnight with a primary antibody (monoclonal rabbit anti-synaptophysin clone EP1098Y, 1:20000 in PBS-BT for males, 1:10000 in PBS-BT for females, Abcam Inc., Cambridge, UK; goat anti-Doublecortin (C18): sc-8066, 1:3000 in PBS-BT, Santa Cruz Biotechnology, Inc., Santa Cruz, CA, USA). After another rinse, the sections were incubated for 90 min with a secondary antibody (donkey anti-rabbit biotinylated IgG, 1:1500 in PBS-BT, Jackson ImmunoResearch, West Grove, PA, USA; donkey-anti-goat biotinylated IgG, 1:1500 in PBS-BT, Jackson ImmunoResearch, West Grove, PA, USA), rinsed again, and incubated with Vector ABC-Elite (A and B, 1:800 in PBS-BT, Vector Laboratories, Burlingame, CA, USA). Finally, the sections were rinsed, preincubated with DAB-Nickel solution, incubated with DAB-Nickel solution with 0.3% H_2_O_2_, and rinsed to stop the reaction. The sections were mounted on gelatin-coated object glasses (0.5% gelatin and 0.05% chrome aluminium sulphate), dried overnight at 37°C, dehydrated in an alcohol series, cleared in Xylol, and enclosed in Entellan. 

### 2.4. Quantification

All quantifications were performed independently by two investigators who were blind to the experimental groups. The mean scores of the two investigators were used in all statistical analyses. 

#### 2.4.1. Synaptophysin

**Figure 1:**
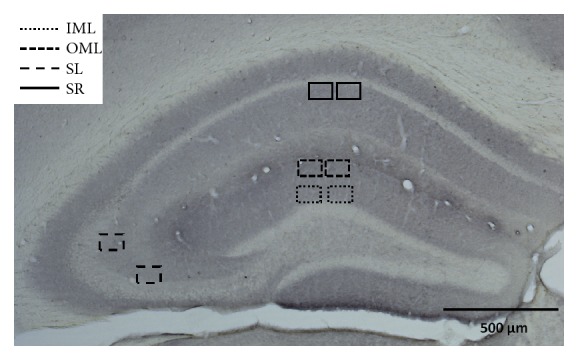
Placement of contours for the analysis of the number of synaptophysin-immunoreactive presynaptic boutons in the hippocampus. The squares indicate the randomly chosen areas in the inner (IML) and outer molecular layer (OML) of the dentate gyrus, the stratum lucidum (SL) of the CA3, and the stratum radiatum (SR) of the CA1.

To determine the number of synaptophysin-immunoreactive presynaptic boutons (SIPBs), appropriate sections were digitised and photomicrographed using a Zeiss Axioskop microscope, equipped with a 100x oil immersion objective and a 10x projection lens. The selection of areas to be quantified was performed with the use of Stereo Investigator (Microbrightfield software, Williston, VT, USA). This software was only used to draw regions of interest and to take pictures. SIPBs were analysed in the prelimbic area (PRL), the cingulate gyrus (GC) and in the CA1, CA3 and dentate gyrus (DG) of the hippocampus (1.6 mm and 0.9 mm anterior and 2.1 mm posterior to bregma, respectively). Within the PRL and GC, two square boxes were placed within the borders of the intended brain areas. In the hippocampus, two randomly chosen regions per section were analysed in the stratum radiatum of area CA1 (SR), stratum lucidum of area CA3 (SL), inner molecular layer (IML), and outer molecular layer (OML) of the DG (Figure 1). Brain regions were based on the mouse brain atlas of Franklin and Paxinos [35]. We have not distinguished between left and right brain regions. In total, four pictures were taken from each brain region (two investigators, two pictures each per brain region). All images taken were then processed with ImageJ (National Institutes of Health) for automatic quantification of the SIPBs. Images were first converted to 8-bit gray scale and then to 16-bit; finally, contrast was enhanced. The threshold was set at 26471–33153 for the males and at 27242–30840 for the females. The thresholds were inevitably different due to differences in the staining sessions. Particles ranging between 0.1 and 4.5 *μ*m^2^ (circularity 0.0-1.0) were considered to be normal-sized SIPBs and included in the analysis. Mean scores per brain area were normalised (WT set as 100%), and these normalised scores were used in the analyses. 

#### 2.4.2. Doublecortin

**Figure 2:**
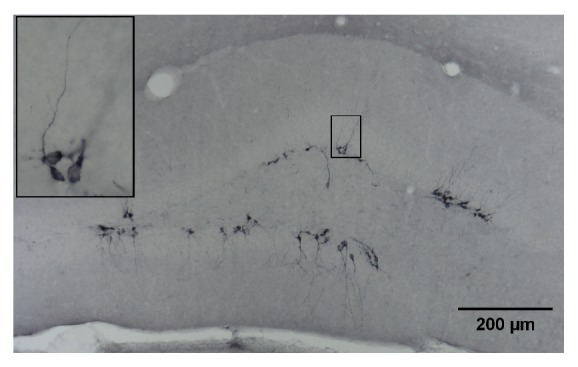
Representative doublecortin-immunostained neurons in the dentate gyrus of the hippocampus (coronal section).

Appropriate sections were digitised and quantified using a Zeiss Axioskop microscope equipped with the Microbrightfield software (Williston, VT, USA). Quantitative analyses were performed with a computer-assisted analysis system (Stereo Investigator). For every mouse, doublecortin-positive cells (Figure 2) were counted in the entire hippocampus in 3 different sections (at 2.1 mm, 2.4 mm, and 2.7 mm posterior to bregma). The brain regions were based on the mouse brain atlas of Franklin and Paxinos [35]. We have not distinguished between left and right brain regions. The contours of the hippocampi were drawn at 5x magnification, and cells were counted at 20x magnification. The mean number of doublecortin-positive newly formed neurons was used in the analysis. 

### 2.5. Statistical Analysis

All statistical analyses were performed with SPSS 16.0. Data were separately analysed for male and female mice with univariate ANOVAs with genotype as the independent factor, followed by Tukey's post hoc HSD. The statistical significance was set at *P* < 0.05.

## 3. Results

### 3.1. Decreased Amount of SIPBs in Female ApoE4 Mice

**Figure 3:**
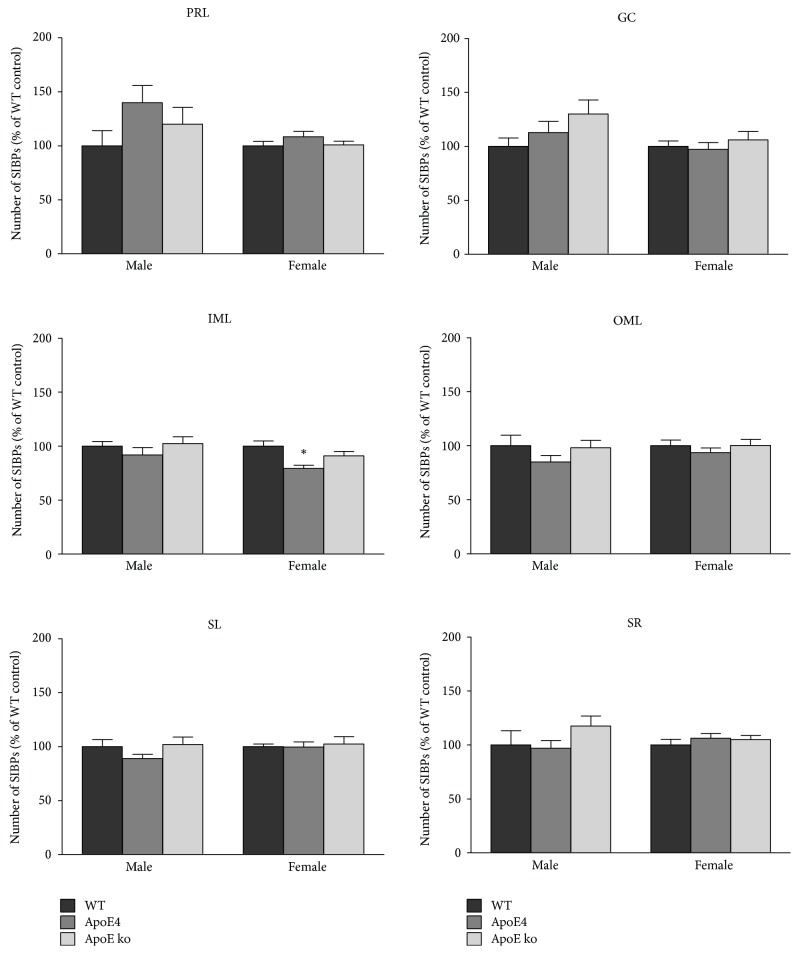
Number of synaptophysin-immunoreactive presynaptic boutons (SIPBs) in the prelimbic area (PRL), the cingulate gyrus (GC), and the stratum radiatum (SR), stratum lucidum (SL) and the inner (IML) and outer molecular layer (OML) of the dentate gyrus in the hippocampus. In female mice, there was a significant effect of genotype on the number of SIPBs in the IML (*n* = 7/group, *P* < 0.01). ApoE4 female mice have less SIPBs than female wild-type mice (∗*P* < 0.01). In other brain regions, no significant differences were found (*n* = 7/group, *P* > 0.05). In male mice, there were no significant effects found in any of the brain regions (*n* = 6–8/group, *P* > 0.05). Error bars show mean ± SEM.

In female mice, an effect of genotype was found on the number of synaptophysin-immunoreactive presynaptic boutons (SIPBs) in the inner molecular layer (IML) of the dentate gyrus in the hippocampus (*F* = 6.884, *P* < 0.01). Post hoc analysis revealed a significant decrease in the number of SIPBs in female apoE4 mice compared with female wild-type (WT) mice (WT: *M* = 100, SEM = 4.67; apoE4: *M* = 79.28, SEM = 2.91; *P* < 0.01, Figure 3). There were no significant differences in the number of SIPBs in the cortex (prelimbic area and cingulate gyrus) or in other regions of the hippocampus (outer molecular layer, stratum radiatum, and stratum lucidum). In male mice, there were no significant differences between the genotypes in the number of SIPBs in any of the investigated brain regions. 

### 3.2. Increased Neurogenesis in Female ApoE ko Mice

**Figure 4:**
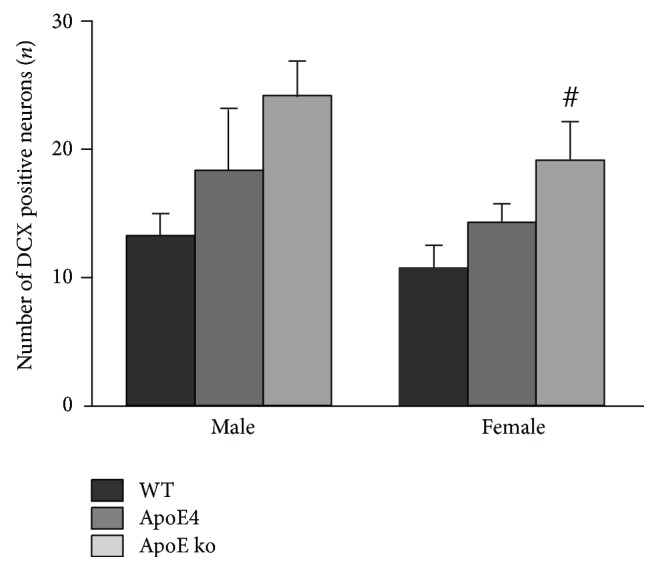
Neurogenesis in the dentate gyrus of the hippocampus. In female mice, a trend was observed in the number of doublecortin-positive newly formed neurons between genotypes (*n* = 6–7 mice/group, ^#^ *P* = 0.052). The data strongly indicate that neurogenesis is increased in female apoE knockout mice compared with female wild-type mice. In males, no differences in neurogenesis were found (*n* = 5–7 mice/group, *P* > 0.05). Error bars show mean ± SEM.

A trend was observed in the number of doublecortin-positive newly formed neurons between genotypes in female mice (*F* = 3.531, *P* = 0.052, Figure 4). There was a strong indication that neurogenesis is increased in female apoE ko mice compared with female WT mice. In male mice, there were no significant differences in neurogenesis between the genotypes.

## 4. Discussion

In this study, we investigated sex differences in apoE, focusing on presynaptic density and neurogenesis, parameters related to cognitive functioning. We studied these parameters in different apoE models of Alzheimer's disease (AD) and vascular disease in both male and female mice. We found a decreased presynaptic density in middle-aged female apoE4 mice and a trend towards increased neurogenesis in middle-aged female apoE knockout (apoE ko) mice compared with wild-type (WT) mice. In male mice, no differences between genotypes could be detected. 

In female apoE4 mice, we found a decrease in the number of synaptophysin-immunoreactive presynaptic boutons (SIPBs) in the inner molecular layer (IML) of the dentate gyrus compared with controls. To our knowledge, no other studies have investigated presynaptic density in aging female apoE4 or apoE ko mice. Studies in male mice either showed no difference between apoE4, apoE ko, and WT mice or demonstrated a decrease in presynaptic density in aged apoE ko mice [36, 37]. The decrease in SIPBs in our female apoE4 mice is in line with autopsy studies in which a decrease in synaptic proteins or synaptic density was found in male and female patients with mild cognitive impairment (MCI) or AD compared with controls [38–40]. However, Scheff et al. [38, 39] did not find a relationship of this decresase with the APOE genotype (*ε*2, *ε*3, *ε*4). 

The fact that we only found an effect in the molecular layer of the dentate gyrus and not in other regions of the hippocampus or in the cortex could indicate that mice at this age are at an early stage of the disease. The molecular layer of the dentate gyrus of the hippocampus receives a direct input from the entorhinal cortex via the perforant pathway [41, 42]. Degeneration of these areas is an early event in both AD and cardiovascular disease [39, 43, 44]. Others have found that the connections between the entorhinal cortex and the molecular layer of the dentate gyrus are selectively vulnerable to synaptic loss [45] in aging humans [39, 46] as well as in several animal models of aging [47, 48]. Neurodegeneration spreads from the entorhinal cortex to the molecular layer of the dentate gyrus and from there to the CA1 and CA3 regions of the hippocampus. Finally, the cerebral cortex, like the prefrontal cortex, is affected when the spreading of neurodegeneration continues. As we only found an effect in the molecular layer of the dentate gyrus and not in the CA1 and CA3 regions or in other cortex regions in our current study, we assume that these middle-aged mice are at an early stage of the disease.

In our female mice, we found the trend of an increased number of doublecortin-positive newly formed neurons, indicating that neurogenesis is enhanced in female apoE ko mice compared with controls. This result contrasts with that of Li et al. [49], who found a decrease in neurogenesis in female apoE ko mice. However, their mice were aged 6-7 months, which is much younger than our 12 to 15-month-old mice. It is possible that at an older age a compensatory mechanism, with an increased number of synapses and/or neurogenesis, is established in response to synaptic failure [50] or insults to the brain [51, 52]. 

In contrast to the female mice, we did not find any differences in presynaptic density or in neurogenesis between male apoE4, apoE ko, and WT mice. The absence of an effect of genotype on the number of SIPBs in male mice is in line with Levi et al. [36], who did not observe differences in presynaptic density in the hippocampus of male apoE3, apoE4, apoE ko, or WT mice. Furthermore, Liraz et al. [53] were not able to detect differences between male apoE3 and apoE4 in hippocampal synaptophysin levels, as determined by western blot. However, Veinbergs et al. [37] did find a decrease in presynaptic density in the hippocampus and frontoparietal cortex of aged male apoE ko mice. In human autopsy studies, male and female patients with AD or MCI are often found to have synaptic loss compared with controls [38–40]. It should be noted however, that the pattern of relative differences between the genotypes for the number of SIPBs is similar for males and females in this study, when looking at the inner and outer molecular layer of the dentate gyrus. Nevertheless, only the decrease in the number of SIPBs in the IML in female apoE4 mice compared with WT controls reaches statistical significance.

Both in AD patients and in AD and apoE mouse models, conflicting results have been found regarding neurogenesis in male sex. Most studies on AD showed a decrease in neurogenesis in both humans [54] and in APP and PS1 mouse models [55, 56]. Conflicting studies found an increase in neurogenesis in male AD patients [52] and in PDGF-APP_Sw_, Ind AD transgenic mice [57]. However, the first study suffers from methodological issues concerning differences between the AD and control group in age, sex, and postmortem interval. In studies of the apoE mouse model, only one reported on male mice. This study by Levi and Michaelson [51] reported that neurogenesis, as measured immunohistochemically by doublecortin staining, is increased in 6-month-old male apoE ko mice and even more in apoE4 mice. In contrast, we did not find any differences in neurogenesis in our male mice. This discrepancy could possibly result from the age difference (6 versus 11 months) between the mice in these studies. It should be noted, however, that visual inspection of the neurogenesis data gives a similar impression for both male and female mice. We cannot exclude the possibility that we would have seen the same trend in male mice with a larger group of animals.

 The present findings support the previously found sex differences between APOE genotypes. It is possible that estrogen plays a role, as it has many effects on the vasculature and on the brain. While estrogen's effects on the vasculature are mainly positive [7, 58–60], its interaction with apoE4 in the brain can have detrimental effects. In vitro regulation of APOE by estrogen is allele-dependent [61], and estradiol does not facilitate neurite outgrowth in the presence of apoE4, while it does so in the presence of apoE2 and apoE3 [33]. Hormone replacement therapy is not beneficial to cognitive function in women who carry at least one *ε*4 allele [31, 32]; however, it can lower the incidence of AD and reduce cognitive decline in women not carrying the *ε*4 allele [62, 63]. In addition, female carriers of an *ε*4 allele, with an increased reproductive period and therefore a longer lifetime exposure to estrogen, have an increased risk for dementia and AD [64]. The harmful effect of estrogen in those carrying the APOE4 genotype could also explain the stronger association of APOE4 with AD in women [26–28] and the increased susceptibility of female apoE4 mice to spatial memory deficits [29, 30]. In contrast, androgens have been shown to protect against apoE4-induced cognitive deficits [65]. Androgen-treated female apoE4 mice improved their performance in a spatial memory test. However, male apoE4 mice, which initially did not show any deficits, were impaired in spatial memory after blockade of androgen receptors. The current result, where only female apoE4 mice show a decreased number of SIPBs, is also consistent with this view of a specific female susceptibility to the negative effect of apoE4. 

In our apoE mouse models, several mechanisms are involved in producing both the decrease in presynaptic boutons in female apoE4 mice and the increase in neurogenesis in female apoE ko mice. The severely compromised vasculature in apoE ko mice (severe atherosclerosis, impaired vascular endothelium-dependent relaxation, and aortic stiffening [66–68]) results in a compensatory effect by increased neurogenesis. The specific interaction of apoE4 with estrogen, even though their vasculature is less affected than that of apoE ko mice (hypercholesterolemia and accelerated atherosclerosis [17]), results in a deleterious effect in the form of a loss of synapses. In addition, male apoE4 mice could possibly benefit from protection by androgens from the effects of apoE4. The fact that a compensatory mechanism can be observed in female apoE ko mice, in contrast to male apoE ko mice, could be due to their difference in estrogen exposure. However, we should interpret the trend of increased neurogenesis in female mice with caution. Although there is no indication of a trend in the male data, visual inspection of the graphs of the neurogenesis data gives a similar impression for both male and female mice. Further research is warranted to clarify this matter.

We found a difference in females compared with males in the number of presynaptic boutons and amount of neurogenesis. Although the slightly higher age of the female group should be kept in mind, we do not expect a large influence due to an age difference of only two months. Because our female mice can be considered premenopausal, large hormonal fluctuations are absent. Therefore, both our male and female mice belong to the same stable age range. 

The C57BL6/J wild-type mice we use in the current study were obtained from Harlan Laboratories, Inc. (Horst, The Netherlands). It is known that these mice carry a gene mutation resulting in an alpha-synuclein gene deletion [69]. Because we obtained the apoE4 and apoE ko mice from different vendors, this could have influenced our results. The alpha-synuclein deletion has some effects on synapse function related to neurotransmitter mobilisation [70–72]. However, basal synaptic transmission is unimpaired [70], and there are no indications of an altered number of (pre)synapses or neurogenesis in C57BL6/alpha-synuclein deletion mice from Harlan. Thus, although we should keep this limitation in mind when interpreting our results, the use of these WT mice is not expected to significantly alter the outcomes of the experiments. 

In the present study, we used the presynaptic marker synaptophysin to obtain information on presynaptic density in apoE4 and apoE ko mice. Measuring SIPBs is only one indicator of the number of synapses. Future studies using postsynaptic markers, such as PSD 95, or electron microscopic evaluation of the number of synapses are needed to acquire a more complete picture of synaptic density in these mouse models.

## 5. Conclusion

To our knowledge, this is the first investigation of presynaptic density in aging female apoE4 and apoE ko mice. We found a decrease in presynaptic density in the hippocampus of middle-aged female apoE4 mice compared with WT mice. This may be the result of a specific harmful interaction of estrogen with apoE4, as we did not observe any differences in male mice. In addition, we found neurogenesis to be increased in middle-aged female apoE ko mice. Previous studies have suggested a compensatory mechanism for synaptic failure by temporarily increasing the number of synaptic contacts and/or neurogenesis. The trend of increased neurogenesis found in female apoE ko mice in our study supports this hypothesis. Our results support the previously determined sex-specific differences observed between APOE genotypes, which could account for some of the sex differences in AD and CVD. Sex differences should be taken into account in any research concerning CVD, AD, or apoE. 

## Abbreviations

A*β*: Amyloid-beta
AD: Alzheimer's disease
ApoE: Apolipoprotein E
APP: Amyloid precursor protein
BBB: Blood brain barrier
CAA: Cerebral amyloid angiopathy
CBF: Cerebral blood flow
CVD: Cardiovascular disease
DG: Dentate gyrus
GC: Cingulate gyrus
ko: Knockout
IML: Inner molecular layer
LDL: Low density lipoprotein
MCI: Mild cognitive impairment
OML: Outer molecular layer
PRL: Prelimbic area
PS: Presenilin
SIPB: Synaptophysin-immunoreactive presynaptic bouton
SL: Stratum lucidum
SR: Stratum radiatum
WT: Wild type.
